# Buffalo Medical Association

**Published:** 1880-05

**Authors:** 


### BUFFALO MEDICAL ASSOCIATION.

THE April meeting of this Association was principally devoted to the election of officers and other business of no special interest professionally.

Judge Beckwith, of this city, however, kindly presented a paper, containing many practical hints concerning the testimony of medical experts—some extracts from which are given in this number of the JOURNAL. Next month more extended selections will be made from that portion, which treats of the right of physicians to demand payment for professional opinions rendered in court.

After the reading of this paper, the Association proceeded to ballot for officers for the ensuing year, the result of which was the election of the following: President, Lucien Howe, M. D.; Vice-President, Albert H. Briggs, M. D.; Secretary, Dougal Macniel, M. D.; Librarian, J. B. Samo, M. D.

